# The Prognostic Value of 14-3-3 Isoforms in Vulvar Squamous Cell Carcinoma Cases: 14-3-3β and ε Are Independent Prognostic Factors for These Tumors

**DOI:** 10.1371/journal.pone.0024843

**Published:** 2011-09-15

**Authors:** Zhihui Wang, Jahn M. Nesland, Zhenhe Suo, Claes G. Trope, Ruth Holm

**Affiliations:** 1 Department of Pathology, The Norwegian Radium Hospital, Oslo University Hospital and University of Oslo, Oslo, Norway; 2 Department of Oncology, The First Affiliated Hospital of Zhengzhou University, Medical College of Zhengzhou University, Zhengzhou, China; 3 Department of Obstetrics and Gynecology, The Norwegian Radium Hospital, Oslo University Hospital and University of Oslo, Oslo, Norway; 4 Department of Pathology, The Norwegian Radium Hospital, Oslo University Hospital, Oslo, Norway; Florida International University, United States of America

## Abstract

**Background:**

The 14-3-3 family is comprised of highly conserved proteins that are functionally important in the maintenance of homeostasis. Their involvement with the cell cycle, their association with proto-oncogenes and oncogenes, and their abnormal expression in various tumors has linked this family of proteins to the etiology of human cancer. Mounting evidence now indicates that 14-3-3σ is a cancer suppressor gene but the roles of the other 14-3-3 isoforms and their interactions in tumorigenesis have not yet been elucidated. In our current study, we examined the expression of 14-3-3β, γ, ε, ζ, η and τ in a large series of vulvar squamous cell carcinomas to evaluate any clinical significance.

**Methods:**

Tumor biopsies from 298 vulvar carcinomas were examined by immunohistochemistry for the expression of 14-3-3β, γ, ε, ζ, η and τ. Statistical analyses were employed to validate any associations between the expression of any 14-3-3 isoform and clinicopathologic variables for this disease.

**Results:**

High cytoplasmic levels of 14-3-3β, γ, ζ, ε and η were observed in 79%, 58%, 50%, 86% and 54% of the vulvar carcinomas analyzed, respectively, whereas a low nuclear expression of 14-3-3τ was present in 80% of these cases. The elevated cytoplasmic expression of 14-3-3β, γ, ε, ζ and η was further found to be associated with advanced disease and aggressive features of these cancers. The overexpression of cytoplasmic 14-3-3β and ε significantly correlated with a poor disease-specific survival by univariate analysis (*P* = 0.007 and *P* = 0.04, respectively). The independent prognostic significance of these factors was confirmed by multivariate analysis (*P* = 0.007 and *P* = 0.009, respectively).

**Conclusions:**

We reveal for the first time that the 14-3-3β, γ, ε, ζ, η and τ isoforms may be involved in the progression of vulvar carcinomas. Furthermore, our analyses show that high cytoplasmic levels of 14-3-3β and ε independently correlate with poor disease-specific survival.

## Introduction

Vulvar squamous cell carcinoma accounts for 3–5% of all gynecological carcinomas [1] and is subcategorized into human papillomavirus (HPV)-independent type and HPV-associated type [2]. The HPV-independent type comprises ∼80% of cases and presents mainly in older patients, whereas the HPV-associated type accounts for ∼20% of these patients and occurs at a comparatively younger age [3]. However, an increasing incidence of these lesions among younger women has been reported recently [4], [5]. Surgery remains the standard treatment for vulvar squamous cell carcinoma and patients with advanced stage diseases are subjected to more extensive and radical surgical interventions, which incurs a higher risk of post-operative complications [6]. Individualized therapy, with the prospect of maximizing the impact of a particular treatment approach, concomitant with fewer complications, is therefore highly desirable in these cases. The identification of new biomarkers of the progression of vulvar carcinomas will greatly assist with the development of such therapies. In this regard, the 14-3-3 protein family warrants particular attention.

The highly conserved 14-3-3 protein family is present in a broad range of organisms [7]. The 14-3-3 proteins are specific phosphoserine/threonine-binding proteins and in humans, seven isoforms (β, γ, ε, ζ, η, σ and τ) have been identified [8]. Their remarkable level of conservation indicates a vital role in cellular physiology [7] and indeed the 14-3-3 proteins interact with a wide variety of cellular molecules via consensus motifs and thereby regulate their activities through different mechanisms [9]. The targets of 14-3-3 include transcription factors, biosynthetic enzymes, cytoskeletal proteins, signaling molecules, apoptosis factors and tumor suppressors [8]. In addition, interactions with multiple proto-oncogene and oncogene products [7] and participation in mitogenic signaling pathways including CDC25 have raised speculations over the roles of the 14-3-3 proteins in the development and progression of malignancy, and also their potential as therapeutic targets [9]. CDC25 phosphatases, which are principal regulators of the entry into mitosis via the activation of CDK1/cyclin B, are themselves regulated spatially and temporally by 14-3-3 in mitogenic signaling pathways [10].

The abnormal expression of 14-3-3 proteins has been reported in various types of carcinoma, including lung [11], gastric [12] and oral carcinoma [13]. Deregulation of some of the 14-3-3 isoforms has also been shown to contribute to tumorigenesis by enhancing tumor aggressiveness [7], [9]. To our knowledge, with the exception of 14-3-3σ, the profile of the 14-3-3 isoforms in vulvar cancers has not been previously reported. We thus investigated the expression profile of these other 14-3-3 isoforms in a large series of vulvar squamous cell carcinomas to evaluate their potential as prognostic indicators of this disease.

## Methods

### Patient materials

A retrospective study was performed on a cohort of 298 patients who had been diagnosed with vulvar squamous cell carcinoma and had undergone a resection at The Norwegian Radium Hospital between 1977 and 2006. The median age at diagnosis was 74 years (range, 35–96 years). Nine of these patients had received other treatments prior to surgery, including six cases that were treated by radiotherapy and three cases that received a combined radiotherapy/chemotherapy intervention. Radical surgery (a total vulvectomy plus a bilateral inguinal lymphadenectomy) was performed in 181 (61%) of these cases and the remaining 117 (39%) patients received non-radical surgery. Postoperative therapy was administered to 70 patients including irradiation in 63 (21%) cases, chemotherapy in three (1%) cases and irradiation/chemotherapy in four (1%) cases. One hundred and twenty-two (41%) of the patients in this cohort died as a result of their vulvar cancer. All patients were followed up from the time of their confirmed diagnosis until death or 1 September, 2009. The median follow-up time was 151 months (range, 43 to 378 months). The tumors were all staged based on the new International Federation of Gynecology and the Obstetrics (FIGO) classification from 2009 [14]. The Regional Committee for Medical Research Ethics South of Norway (S-06012), The Social and Health Directorate (04/2639 and 06/1478) and The Data Inspectorate (04/01043) approved the current study protocol.

All histological specimens were reviewed by J.M.N. (a co-author) without access to any clinical information on the patients and tumor classifications adhered strictly to the World Health Organization recommendations [15]. Two hundred and eighty (94%) tumors were classified as keratinizing/non-keratinizing, 14 (5%) as basaloid and four (1%) as veruccous. Control samples of 10 normal vulva tissues from patients with benign gynecological diseases were also collected following surgery. Results from our previous studies on 14-3-3σ and CDC25s expression in this same cohort of vulvar carcinomas [16], [17] were co-analyzed with those of the current study.

### Immunohistochemistry

Formalin-fixed, paraffin-embedded sections were processed for immunohistochemistry using the Dako EnVision™ Flex+ System (K8012; Dako, Glostrup, Denmark) and the Dako Autostainer. Deparaffinization and the unmasking of epitopes were performed using PT-Link (Dako) and EnVision™ Flex target retrieval solution at a low pH. To block endogenous peroxidase activity, the sections were treated with 0.03% hydrogen peroxide (H_2_O_2_) for 5 min. The sections were then incubated for 30 min with polyclonal rabbit antisera raised against 14-3-3β (AHP1044, 1∶1000), 14-3-3γ (AHP1047, 1∶2000), 14-3-3ζ (AHP1052, 1∶2000), 14-3-3ε (AHP1048, 1∶1000), 14-3-3η (AHP1046, 1∶3000) or 14-3-3τ (AHP1051, 1∶1000), all of which were purchased from AbD Serotec (Oxford, UK). The primary antibodies against the 14-3-3β and ζ isoforms showed low cross-reactivity with 14-3-3ζ and β, respectively, whereas all other 14-3-3 primary antibodies showed a high degree of specificity [18].

Following the antibody treatments, the slides were incubated with EnVision™ Flex+ rabbit linker (15 min) and EnVision™ Flex/HRP enzyme (30 min). The tissues were subsequently stained for 10 minutes with 3′3-diaminobenzidine tetrahydrochloride (DAB) and then counterstained with hematoxylin. The samples were then dehydrated and mounted in Diatex. All of the sample series included appropriate positive controls, which included cervical cancer (14-3-3β), ovarian cancer (14-3-3ε and ζ), colon cancer (14-3-3η and γ) and prostate cancer (14-3-3τ) sections. As negative controls, the polyclonal rabbit antisera were replaced with normal rabbit serum at the equivalent dilution.

A semi-quantitative scale was used to score the immunohistochemical signals detected in the cancer cells on each section both in the cytoplasm and the nucleus. The scores (range, 0 to 9) were individually produced by multiplying the value for the intensity of the signal (absent, 0; weak, 1; moderate, 2; strong, 3) by the extent of the immunoreactivity (i.e. the percentage of positive cancer cells: absent, 0; <10%, 1; 10–50%, 2; >50%, 3). The cutoff value for the immunoreactivity of each 14-3-3 isoform in the cytoplasm or nucleus was chosen based on the individual staining pattern for each in the normal vulvar epithelium. High cytoplasmic immunostaining for the different isoforms was classified as follows: score >1 for 14-3-3β and ε, score >3 for 14-3-3γ, ζ and η and score >0 for 14-3-3τ. High nuclear immunostaining for the isoforms was assigned as follows: score >0 for 14-3-3β, γ and η and score ≥6 for 14-3-3ζ, ε and τ. The immunostaining results were evaluated independently by two observers (Z.W. and R.H.) with no prior knowledge of the patient outcomes. All discordant scores were reviewed until a final agreement was obtained.

### Statistical analyses

The Pearson's chi-square (χ^2^) test was used to find associations between the 14-3-3 isoform expression patterns and the clinicopathologic variables. The disease-specific survival analysis was processed using the Kaplan and Meier estimation and log-rank test. A Cox proportional hazards regression model was used for further analyses of the univariate and multivariate evaluations of survival rates. In the multivariate analysis, a backward stepwise regression was executed among variables that were eligible with a *P*≤0.05 in the univariate analysis. All analyses were processed using the SPSS 16.0 statistical software package (SPSS, Chicago, IL). Statistical significance was considered for *P*≤0.05.

## Results

### 14-3-3 protein expression

**Figure 1:**
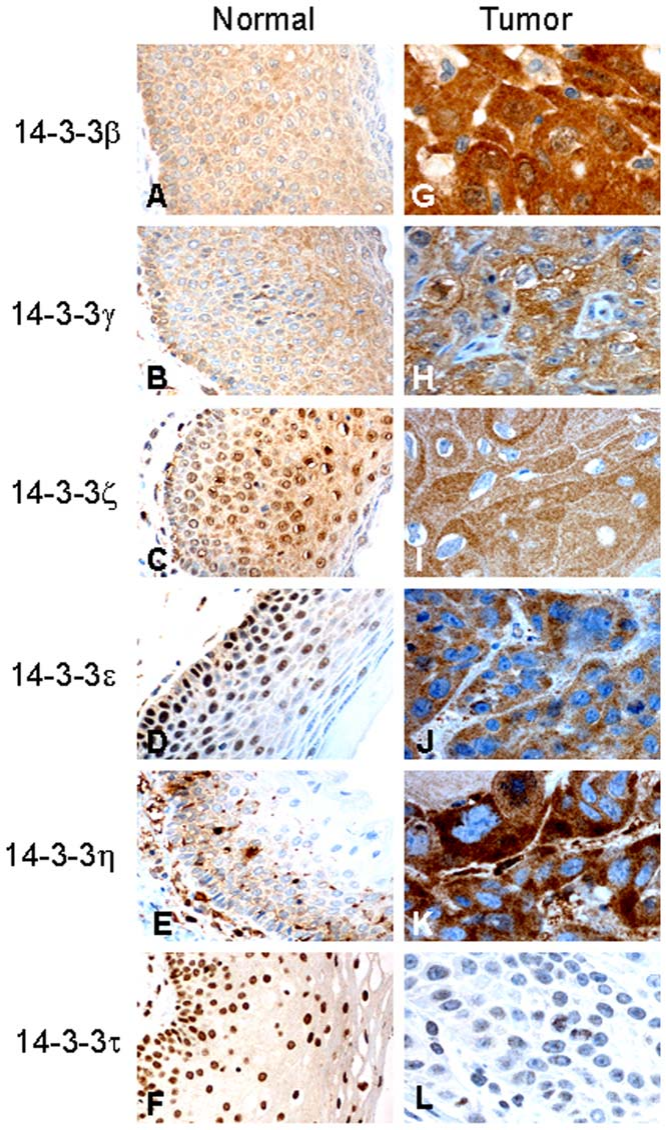
The expression of 14-3-3 proteins in the vulvar squamous epithelium. Representative samples stained using immunohistochemistry are shown. The expression of the 14-3-3 proteins (β, γ, ζ, ε, η and τ) in normal vulvar squamous epithelium (A–F). The increased cytoplasmic expression of 14-3-3β, γ, ζ, ε and η (G–K) and the reduced nuclear expression of 14-3-3τ (L) in vulvar carcinomas.

In 10 cases of normal vulvar squamous epithelium the cytoplasmic staining was observed for 14-3-3β (score ≤3), 14-3-3γ (score 3), 14-3-3ζ (score 3) and 14-3-3ε (score ≤3) in the basal, parabasal, middle and top layers, whereas cytoplasmic staining for 14-3-3η (score 3) was limited to the basal, parabasal and middle layers. Nuclear staining for 14-3-3ζ (score ≥6), 14-3-3ε (score 6) and 14-3-3τ (score ≥6) was detected as a constant distribution from the basal to top layers (Figures 1A–F). The subcellular immunostaining assessment of each 14-3-3 isoform in the vulvar carcinoma series under study is summarized in Table S1. In the cytoplasm, a high expression of 14-3-3β (score >1), 14-3-3γ (score >3), 14-3-3ζ (score >3), 14-3-3ε (score >1) and 14-3-3η (score >3) was observed in 79% (236/298), 58% (172/298), 50% (150/298), 86% (256/298) and 54% (160/298) of the patients, respectively (Figures 1G-K). In the nucleus, a high expression of 14-3-3β, γ and η (all score >0) was observed in 8% (24/298), 15% (47/298) and 1% (3/298) of the cases, respectively. A low signal for 14-3-3ζ and ε (both score <6) in the nucleus was found in 97% (289/298) and 98% (291/298) of the cases, respectively. Low nuclear expression of 14-3-3τ (score <6) was also found in 80% (239/298) of the patients (Figure 1L).

### Association of the 14-3-3 isoform immunostaining profiles with the clinicopathological parameters and CDC25 isoform status

The expression patterns of all of the 14-3-3 isoforms (nuclear staining for τ and cytoplasmic expression for all others) were found to correlate with the tumor diameter in the vulvar squamous cell carcinoma patient subjects. The expression of 14-3-3β, γ, ζ, ε and η correlated with the depth of tumor invasion, whereas the expression of 14-3-3γ and ζ correlated with the FIGO substage of these cancers. Moreover, the expression of 14-3-3γ correlated with the advancement of lymph node metastasis and the expression of 14-3-3η correlated with the tumor differentiation. None of the 14-3-3 isoforms showed any association with the patient's age or with vessel infiltration (Table S2). The expression patterns of the 14-3-3β, γ, ζ, ε and η isoforms in the nucleus were also not found to significantly correlate with any of the clinicopathological parameters tested for the patient cohort. The expression of all of the 14-3-3 isoforms (in the nucleus for τ and in the cytoplasm for all others) correlated with that of phospho-CDC25C (Ser216). Further, the expression of 14-3-3γ, ε, ζ and η correlated with that of CDC25C and the expression of 14-3-3β, ε, ζ, η and τ with that of CDC25A. The expression of 14-3-3γ correlated with that of CDC25B (Table S3). Moreover, the expression of 14-3-3β, γ, ζ, ε and η correlated significantly with all other 14-3-3 isoforms with the exception of 14-3-3τ (Table S3).

**Figure 2:**
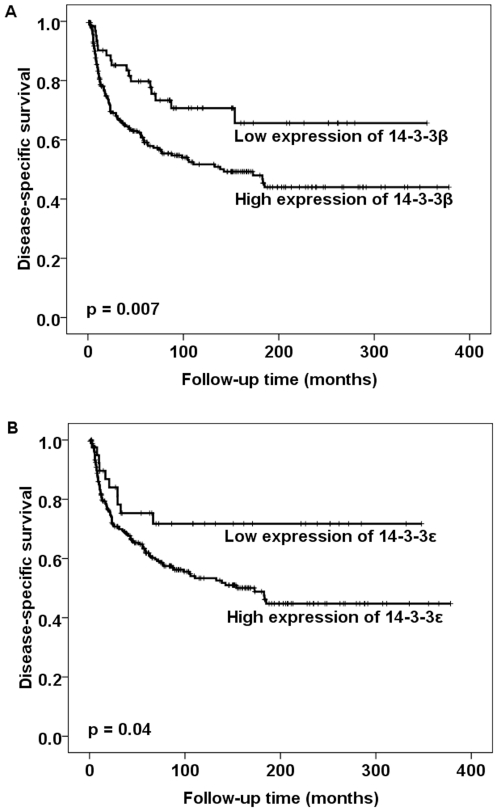
Survival curves using the Kaplan-Meier method. The Kaplan-Meier curve of disease-specific survival in relation to the cytoplasmic expression of 14-3-3β and ε. The *p*-value for 14-3-3β differs slightly from that in Table 1 based on the use of the log-rank test in Figure 2 as opposed to the use of the Cox-regression analysis in Table 1.

By univariate analysis, the high cytoplasmic expression of 14-3-3β and ε was found to be associated with a poor disease-specific survival outcome (Figure 2). By multivariate analysis, FIGO, lymph node metastasis, age, vessel infiltration and the cytoplasmic expression of 14-3-3β and ε were found to retain their independent prognostic significance for vulvar squamous cell carcinoma (Table 1).

**Table 1:** Relative risk (RR) of dying from vulvar cancer.

Variables	Univariate analysis			Multivariate analysis		
	RR	95% CI^a^	*P*	RR	95% CI^a^	*P*
FIGO	1.51	1.38–1.65	<0.001	1.29	1.12–1.49	0.001
Lymph node metastasis	2.45	1.90–3.14	<0.001	1.84	1.33–2.53	<0.001
Age	1.62	1.24–2.12	<0.001	1.68	1.20–2.34	0.002
Infiltration of vessel	2.47	1.69–3.61	<0.001	1.82	1.17–2.83	0.008
Tumor diameter	1.75	1.38–2.21	<0.001	-	-	-
14-3-3β cytoplasmic staining^b^	2.00	1.19–3.32	0.009	2.42	1.27–4.60	0.007
14-3-3ε cytoplasmic staining^b^	1.96	1.02–3.74	0.04	3.40	1.36–8.46	0.009

a 95% confidence interval.

b Cytoplasma: low ≤1 and high >1.

## Discussion

Compared with the expression of 14-3-3 isoforms in normal vulvar tissues, the higher cytoplasmic expression of 14-3-3β, γ, ζ, ε and η, the higher nuclear expression of 14-3-3β, γ and η, and the lower nuclear expression of 14-3-3ζ and ε were observed in our current analyses of a vulvar carcinoma patient sample series. Our results indicated that the expression of 14-3-3τ is restricted to the nucleus and is downregulated in 80% of the vulvar cancer cases in our cohort when compared with normal tissue. The elevated cytoplasmic expression of 14-3-3β, γ, ζ, ε and η was found to be associated with advanced disease and aggressive tumor characteristics, including a high FIGO substage, the presence of lymph node metastases, large tumor diameters, deeper invasiveness and/or a low histologic grade. Furthermore, our current findings revealed that the high cytoplasmic levels of 14-3-3β and ε significantly correlated with a poor disease-specific survival outcome for vulvar squamous cell carcinoma by both univariate and multivariate analysis.

The expression of 14-3-3β and ε and its association with patient outcomes have not been previously investigated for any human cancer. In our present study, we show that high cytoplasmic levels of both 14-3-3β and ε significantly correlate with a shorter survival time for patients with vulvar carcinoma. Pathogenic roles of 14-3-3β and ε have been previously reported in several studies. The exogenous expression of 14-3-3β increased the proliferation of NIH3T3 cells, their colony formation in soft agar, and tumor formation in nude mouse xenograft experiments [7]. In hepatoma cells, the downregulation of 14-3-3β by antisense 14-3-3β RNA was found to suppress tumor cell growth both in vitro and in vivo [19]. Reduced 14-3-3β levels were found previously to promote a decrease in VEGF production, inhibit angiogenesis, increase apoptosis and suppress tumor size [20]. Furthermore, the upregulation of 14-3-3ε has been demonstrated to play an important role in the development of human renal carcinoma [21] and meningioma [22]. In our current experiments, elevated 14-3-3β and ε significantly correlated with a large tumor diameter and enhanced invasiveness. Cao et al. [23] and Liu et al. [22] have reported in earlier studies of human astrocytomas and meningiomas, respectively, that the expression of 14-3-3β is markedly increased concomitantly with an increase in the pathological grade. A further study of larynx carcinomas has found that the protein levels of 14-3-3ε are significantly lower in stage III or IV tumors compared with stage I or II lesions [24]. Taken together, these findings suggest that 14-3-3β and ε are involved in the progression of vulvar carcinomas and may predictive markers of the survival outcomes in affected patients. Further studies will be needed to confirm the potential of 14-3-3β and ε as prognostic markers of cancer in a clinical setting.

Our present results show a high expression of the 14-3-3γ, ζ and η isoforms in the cytoplasm of a high proportion of vulvar carcinoma samples and a significantly correlation between this expression profile and malignancy in these cancers, including a large tumor diameter and increased invasiveness. 14-3-3γ and ζ expression also significantly correlated with a high FIGO substage, and the 14-3-3γ levels further correlated with presence of lymph node metastases and that 14-3-3η correlated with more poorly differentiated tumor cells. In agreement with our current findings, several earlier studies have reported the upregulation of 14-3-3γ and ζ in different tumor types. Elevated 14-3-3γ expression has been described in lung cancer [25], [26] and in some papillomavirus-induced carcinomas [27]. A high level of 14-3-3ζ has also been found in oral squamous cell carcinoma [13], stomach cancer [12], breast cancer [28] and meningioma [22]. Few studies have investigated the upregulation of 14-3-3η in cancer. However, Cao et al. [23] have previously demonstrated a high level of 14-3-3η in human astrocytomas. In line with our current findings also, 14-3-3η expression has been shown to be markedly augmented in parallel with an increased pathological grade in human astrocytomas [23]. We speculate therefore that the increased expression of the 14-3-3γ, ζ and η isoforms may be involved in the carcinogenesis of different tumors, including vulvar carcinoma. This speculation is strengthened by the findings of previous reports showing that exogenous 14-3-3γ leads to polyploidization in H322 lung cancer cells [25], that the overexpression of 14-3-3γ in a lung cancer cell line promotes re-entry into S phase [26], and that the overexpression of 14-3-3ζ induces the proliferation of breast cancer cells [28]. Furthermore, our current results suggest that elevated 14-3-3γ, ζ and η expression play an important role in the progression of vulvar carcinomas, despite their limited value as predictors of survival outcomes.

The low expression of 14-3-3τ in the nucleus was identified in 80% of vulvar cancer cases in our present patient cohort when compared to its high levels in normal vulvar tissues. In addition, the low nuclear expression of this isoform significantly correlated with larger tumor diameters but not with disease-specific survival. Our results thus suggest that the downregulation of 14-3-3τ may be involved in the tumorigenesis of vulvar cancer. In contrast, Martin et al. [29] have reported that the increased expression of 14-3-3τ enhances the growth rate of mammary carcinoma cells by promoting cell adhesion to tenascin-C, an extracellular matrix protein. These differences in the effects of the 14-3-3τ protein may indicate tumor type-specific roles.

Our results additionally show that the high cytoplasmic expression of 14-3-3β, γ, ε, ζ and η significantly correlates with all other 14-3-3 isoforms with the exception of 14-3-3τ. This suggests that both homo- and hetero-dimer complexes of 14-3-3 isoforms are formed in vulvar carcinomas. Little is known about the interactions between 14-3-3 isoforms in the cell but these proteins have been found to form thermodynamically stable homo- or hetero-dimers [30]. For example, 14-3-3γ forms homodimers as well as heterodimers with 14-3-3ε. In turn, 14-3-3ε may preferentially form heterodimers rather than homodimers with another family member such as the β, γ, ζ and η isoforms [30]. By forming dimers, 14-3-3 proteins can adopt a wide variety of tertiary structures to bind different protein ligands, which accounts for the multiple roles of these proteins in the regulation of a wide variety of cellular processes [30].

We observed a significant correlation between cytoplasmic 14-3-3β, ε, ζ and η and nuclear CDC25A, as well as between cytoplasmic 14-3-3γ and nuclear CDC25B. Because 14-3-3 and CDC25 isoforms localize in different subcellular regions, these significant correlations indicate that indirect connections occur between these isoforms. The cytoplasmic overexpression of CDC25C and phospho-CDC25C (Ser216) was found in our analysis to correlate with a high cytoplasmic expression of 14-3-3γ, ε and η. Previously, a number of studies reported that the phosphorylation of Ser216 on CDC25C was required for its binding to 14-3-3 [31]-[33]. Telles et al. [31] have further reported that the unique secondary structure of 14-3-3ε, as indicated by the Phe135 residue, and 14-3-3γ, due to an E94-K142 salt bridge, are required to form the 14-3-3ε/γ-CDC25C heterodimer, which inhibits CDC25C function. We suggest from our present data that in vulvar carcinomas, 14-3-3γ and ε are the two most likely isoforms to form complexes with phospho-CDC25C (Ser216) and to thereby regulate CDC25C function. This complex has been found to sequester CDC25C in the cytoplasm, which prevents its binding to CDK1/cyclin B [10]. Hence, this complex serves to negatively regulate the cell cycle. Interestingly, our previous analysis of the same cohort of vulvar carcinoma cases revealed that 70% of the tumors displayed a high nuclear expression of phospho-CDC25C (Ser216) [17]. This phenomenon may be explained by the relatively lower levels of 14-3-3γ and ε compared with those of phospho-CDC25C (Ser216) in these lesions. Once the binding capacity of 14-3-3 and phospho-CDC25C (Ser216) is saturated, unbound CDC25C (Ser216) accumulates in the nucleus, which activates CDK1/cyclin B and thus initiate mitosis [10].

The staining pattern of 14-3-3 isoforms in normal vulvar tissues differs from that in vulvar carcinomas. The regulation of the distribution of each 14-3-3 isoform during tumorigenesis and tumor progression remains unclear however. Previously, intracellular trafficking of 14-3-3 via its nuclear export signal (NES), a leucine-rich region within the COOH-terminal, has been reported to shuttle 14-3-3 proteins in and out of the nucleus [9], [34]. However, there is no consensus in the current literature regarding the function of this region. According to Brunet et al. [34] and Gardino et al. [35], the NES of 14-3-3 is a ligand binding region rather than a motif that functions in nuclear signaling transport. This suggests that the binding of a ligand to the NES of 14-3-3 may regulate the subcellular trafficking of this ligand. Hence, the specific subcellular locations of the 14-3-3 isoforms may be independently regulated by their corresponding ligands.

In conclusion, the abnormal expression of 14-3-3β, γ, ε, ζ, η and τ in a high proportion of vulvar carcinomas and the correlations of these levels with the clinicopathological features of these tumors indicates that 14-3-3 isoforms may be involved in the progression of vulvar carcinomas. Furthermore, 14-3-3β and ε are independent predictors of poor disease-specific survival in cancer patients due to the high cytoplasmic levels of these proteins in cancer cells.

## Supporting Information

Table S1 **Expression of 14-3-3 isoforms in 298 vulvar carcinomas assessed by immunohistochemistry.**
(DOC)

Table S2 **14-3-3 isoform expression in relation to clinicopathological variables in vulvar carcinomas.**
(DOC)

Table S3 **14-3-3 isoform expression in relation to CDC25 isoforms in vulvar carcinomas.**
(DOC)
