# Supplementary material for: The Prognostic Value of 14-3-3 Isoforms in Vulvar Squamous Cell Carcinoma Cases: 14-3-3β and ε Are Independent Prognostic Factors for These Tumors
Source: PLoS One. 2011 Sep 15;6(9):e24843. doi: 10.1371/journal.pone.0024843 (PMC3174199; doi:10.1371/journal.pone.0024843)
Supplement: Table S1 — Expression of 14-3-3 isoforms in 298 vulvar carcinomas assessed by immunohistochemistry. (DOC) [file pone.0024843.s001.doc]

Table S1. Expression of 14-3-3 isoforms in 298 vulvar carcinomas assessed by immunohistochemistry

| Score | 14-3-3β | | | |  | 14-3-3γ | | | |  | 14-3-3ζ | | | |  | 14-3-3ε | | | |  | 14-3-3η | | | |  | 14-3-3τ | | | |
| --- | --- | --- | --- | --- | --- | --- | --- | --- | --- | --- | --- | --- | --- | --- | --- | --- | --- | --- | --- | --- | --- | --- | --- | --- | --- | --- | --- | --- | --- |
|  | Cyto | | Nuc | |  | Cyto | | Nuc | |  | Cyto | | Nuc | |  | Cyto | | Nuc | |  | Cyto | | Nuc | |  | Cyto | | Nuc | |
|  | n | (%) | n | (%) |  | n | (%) | n | (%) |  | n | (%) | n | (%) |  | n | (%) | n | (%) |  | n | (%) | n | (%) |  | n | (%) | n | (%) |
| 0 | 55 | (19) | 274 | (92) |  | 19 | (6) | 251 | (84) |  | 62 | (21) | 226 | (76) |  | 36 | (12) | 202 | (68) |  | 21 | (7) | 295 | (99) |  | 298 | (100) | 19 | (6) |
| 1 | 7 | (2.3) | 0 | (0) |  | 3 | (1) | 1 | (0.3) |  | 1 | (0.3) | 41 | (14) |  | 6 | (2) | 1 | (0.3) |  | 0 | (0) | 0 | (0) |  | 0 | (0) | 14 | (5) |
| 2 | 44 | (15) | 13 | (4) |  | 57 | (19) | 21 | (7) |  | 33 | (11) | 7 | (2) |  | 55 | (19) | 47 | (16) |  | 45 | (15) | 2 | (0.7) |  | 0 | (0) | 92 | (31) |
| 3 | 58 | (20) | 4 | (1) |  | 47 | (16) | 5 | (2) |  | 52 | (17) | 15 | (5) |  | 65 | (22) | 8 | (3) |  | 72 | (24) | 0 | (0) |  | 0 | (0) | 24 | (8) |
| 4 | 42 | (14) | 5 | (2) |  | 38 | (13) | 15 | (5) |  | 47 | (16) | 0 | (0) |  | 53 | (18) | 33 | (11) |  | 51 | (17) | 0 | (0) |  | 0 | (0) | 90 | (30) |
| 6 | 72 | (24) | 2 | (1) |  | 83 | (28) | 5 | (2) |  | 76 | (26) | 9 | (3) |  | 71 | (24) | 6 | (2) |  | 85 | (29) | 1 | (0.3) |  | 0 | (0) | 52 | (17) |
| 9 | 20 | (7) | 0 | (0) |  | 51 | (17) | 0 | (0) |  | 27 | (9) | 0 | (0) |  | 12 | (4) | 1 | (0.3) |  | 24 | (8) | 0 | (0) |  | 0 | (0) | 7 | (2) |

Cyto = Cytoplasm

Nuc = Nucleus
