# Supplementary material for: The Prognostic Value of 14-3-3 Isoforms in Vulvar Squamous Cell Carcinoma Cases: 14-3-3β and ε Are Independent Prognostic Factors for These Tumors
Source: PLoS One. 2011 Sep 15;6(9):e24843. doi: 10.1371/journal.pone.0024843 (PMC3174199; doi:10.1371/journal.pone.0024843)
Supplement: Table S2 — 14-3-3 isoform expression in relation to clinicopathological variables in vulvar carcinomas. (DOC) [file pone.0024843.s002.doc]

Table S2. 14-3-3 isoform expression in relation to clinicopathological variables in vulvar carcinomas

| Variables |  | 14-3-3β | | |  | 14-3-3γ | | |  | 14-3-3ζ | | |  | 14-3-3ε | | |  | 14-3-3η | | |  | 14-3-3τ | | |
| --- | --- | --- | --- | --- | --- | --- | --- | --- | --- | --- | --- | --- | --- | --- | --- | --- | --- | --- | --- | --- | --- | --- | --- | --- |
|  |  | Cytoplasm | | |  | Cytoplasm | | |  | Cytoplasm | | |  | Cytoplasm | | |  | Cytoplasm | | |  | Nucleus | | |
|  | n | H | (%) | *P*^1^ |  | H | (%) | *P* |  | H | (%) | *P* |  | H | (%) | *P* |  | H | (%) | *P* |  | L | (%) | *P* |
| Age |  |  |  | 0.69 |  |  |  | 0.62 |  |  |  | 0.47 |  |  |  | 0.40 |  |  |  | 0.13 |  |  |  | 0.65 |
| 25-69 | 118 | 95 | (81) |  |  | 64 | (54) |  |  | 63 | (53) |  |  | 98 | (83) |  |  | 57 | (48) |  |  | 97 | (82) |  |
| 70-84 | 146 | 116 | (80) |  |  | 88 | (60) |  |  | 73 | (50) |  |  | 127 | (87) |  |  | 80 | (55) |  |  | 114 | (78) |  |
| 85+ | 34 | 25 | (74) |  |  | 20 | (59) |  |  | 14 | (41) |  |  | 31 | (91) |  |  | 23 | (68) |  |  | 28 | (83) |  |
| FIGO |  |  |  | 0.21 |  |  |  | 0.02 |  |  |  | 0.03 |  |  |  | 0.14 |  |  |  | 0.10 |  |  |  | 0.24 |
| Ia | 10 | 6 | (60) |  |  | 6 | (60) |  |  | 4 | (40) |  |  | 9 | (90) |  |  | 3 | (30) |  |  | 8 | (80) |  |
| Ib | 34 | 23 | (68) |  |  | 11 | (32) |  |  | 10 | (29) |  |  | 26 | (77) |  |  | 10 | (29) |  |  | 26 | (77) |  |
| II | 117 | 91 | (78) |  |  | 61 | (52) |  |  | 63 | (54) |  |  | 104 | (89) |  |  | 68 | (58) |  |  | 97 | (83) |  |
| IIIa | 64 | 52 | (81) |  |  | 45 | (70) |  |  | 28 | (44) |  |  | 52 | (81) |  |  | 36 | (56) |  |  | 45 | (70) |  |
| IIIb | 38 | 34 | (90) |  |  | 25 | (66) |  |  | 25 | (66) |  |  | 35 | (92) |  |  | 22 | (58) |  |  | 34 | (90) |  |
| IIIc | 12 | 12 | (100) |  |  | 8 | (67) |  |  | 8 | (67) |  |  | 12 | (100) |  |  | 7 | (58) |  |  | 11 | (92) |  |
| IVa | 5 | 4 | (80) |  |  | 3 | (60) |  |  | 1 | (20) |  |  | 3 | (60) |  |  | 2 | (40) |  |  | 3 | (60) |  |
| IVb | 13 | 10 | (77) |  |  | 9 | (69) |  |  | 7 | (54) |  |  | 10 | (77) |  |  | 9 | (69) |  |  | 10 | (77) |  |
| Not available | 5 |  |  |  |  |  |  |  |  |  |  |  |  |  |  |  |  |  |  |  |  |  |  |  |
| Lymph node metastasis |  |  |  | 0.09 |  |  |  | 0.01 |  |  |  | 0.69 |  |  |  | 0.18 |  |  |  | 0.29 |  |  |  | 0.15 |
| None | 138 | 103 | (75) |  |  | 70 | (51) |  |  | 67 | (49) |  |  | 121 | (88) |  |  | 68 | (49) |  |  | 112 | (81) |  |
| Unilateral | 83 | 72 | (87) |  |  | 57 | (69) |  |  | 44 | (53) |  |  | 74 | (89) |  |  | 50 | (60) |  |  | 63 | (76) |  |
| Bilateral | 34 | 28 | (82) |  |  | 24 | (71) |  |  | 19 | (56) |  |  | 26 | (77) |  |  | 18 | (53) |  |  | 31 | (91) |  |
| Not available | 43 |  |  |  |  |  |  |  |  |  |  |  |  |  |  |  |  |  |  |  |  |  |  |  |
| Tumor diameter (cm) |  |  |  | 0.01 |  |  |  | <0.001 |  |  |  | 0.001 |  |  |  | 0.02 |  |  |  | <0.001 |  |  |  | 0.03 |
| 0.3-2.5 | 88 | 62 | (71) |  |  | 35 | (40) |  |  | 32 | (37) |  |  | 69 | (78) |  |  | 34 | (39) |  |  | 64 | (73) |  |
| 2.6-4.0 | 94 | 74 | (79) |  |  | 56 | (60) |  |  | 52 | (55) |  |  | 82 | (87) |  |  | 49 | (52) |  |  | 82 | (87) |  |
| 4.1-20.0 | 100 | 88 | (88) |  |  | 75 | (75) |  |  | 63 | (63) |  |  | 92 | (92) |  |  | 68 | (68) |  |  | 83 | (83) |  |
| Not available | 16 |  |  |  |  |  |  |  |  |  |  |  |  |  |  |  |  |  |  |  |  |  |  |  |
| Tumor differentiation |  |  |  | 0.37 |  |  |  | 0.20 |  |  |  | 0.06 |  |  |  | 1 |  |  |  | 0.002 |  |  |  | 0.43 |
| Well | 73 | 55 | (75) |  |  | 38 | (52) |  |  | 28 | (38) |  |  | 63 | (86) |  |  | 27 | (37) |  |  | 57 | (78) |  |
| Moderate | 154 | 121 | (79) |  |  | 87 | (57) |  |  | 84 | (55) |  |  | 132 | (86) |  |  | 87 | (57) |  |  | 128 | (83) |  |
| Poor | 71 | 60 | (85) |  |  | 47 | (66) |  |  | 38 | (54) |  |  | 61 | (86) |  |  | 46 | (65) |  |  | 54 | (76) |  |
| Depth of invasion (mm) |  |  |  | 0.001 |  |  |  | <0.001 |  |  |  | <0.001 |  |  |  | 0.004 |  |  |  | 0.01 |  |  |  | 0.21 |
| 0.0-4.0 | 77 | 53 | (69) |  |  | 24 | (31) |  |  | 20 | (26) |  |  | 58 | (75) |  |  | 31 | (40) |  |  | 60 | (78) |  |
| 4.1-8.0 | 98 | 74 | (76) |  |  | 62 | (63) |  |  | 57 | (58) |  |  | 89 | (91) |  |  | 53 | (54) |  |  | 77 | (79) |  |
| 8.1-40.0 | 122 | 101 | (90) |  |  | 80 | (71) |  |  | 71 | (63) |  |  | 101 | (90) |  |  | 70 | (63) |  |  | 97 | (87) |  |
| Not available | 11 |  |  |  |  |  |  |  |  |  |  |  |  |  |  |  |  |  |  |  |  |  |  |  |
| Infiltration of vessel |  |  |  | 0.73 |  |  |  | 0.67 |  |  |  | 0.33 |  |  |  | 1 |  |  |  | 1 |  |  |  | 0.86 |
| No | 230 | 181 | (79) |  |  | 130 | (57) |  |  | 119 | (52) |  |  | 197 | (86) |  |  | 123 | (54) |  |  | 183 | (80) |  |
| Yes | 65 | 53 | (82) |  |  | 39 | (60) |  |  | 29 | (45) |  |  | 56 | (86) |  |  | 35 | (54) |  |  | 53 | (81) |  |
| Not available | 3 |  |  |  |  |  |  |  |  |  |  |  |  |  |  |  |  |  |  |  |  |  |  |  |

^1^ Pearson chi-square

H = High

L = Low
