# Supplementary material for: The Prognostic Value of 14-3-3 Isoforms in Vulvar Squamous Cell Carcinoma Cases: 14-3-3β and ε Are Independent Prognostic Factors for These Tumors
Source: PLoS One. 2011 Sep 15;6(9):e24843. doi: 10.1371/journal.pone.0024843 (PMC3174199; doi:10.1371/journal.pone.0024843)
Supplement: Table S3 — 14-3-3 isoform expression in relation to CDC25 isoforms in vulvar carcinomas. (DOC) [file pone.0024843.s003.doc]

Table S3. 14-3-3 isoform expression in relation to CDC25 isoforms in vulvar carcinomas

| Variables |  | 14-3-3β | | |  | 14-3-3γ | | |  | 14-3-3ζ | | |  | 14-3-3ε | | |  | 14-3-3η | | |  | 14-3-3τ | | |
| --- | --- | --- | --- | --- | --- | --- | --- | --- | --- | --- | --- | --- | --- | --- | --- | --- | --- | --- | --- | --- | --- | --- | --- | --- |
|  |  | Cytoplasm | | |  | Cytoplasm | | |  | Cytoplasm | | |  | Cytoplasm | | |  | Cytoplasm | | |  | Nucleus | | |
|  | n | H | (%) | *P*^1^ |  | H | (%) | *P* |  | H | (%) | *P* |  | H | (%) | *P* |  | H | (%) | *P* |  | L | (%) | *P* |
| CDC25A nucleus^2^ |  |  |  | 0.03 |  |  |  | 0.06 |  |  |  | 0.001 |  |  |  | 0.01 |  |  |  | 0.02 |  |  |  | 0.02 |
| Low (< 9) | 147 | 124 | (84) |  |  | 93 | (63) |  |  | 88 | (60) |  |  | 134 | (91) |  |  | 89 | (61) |  |  | 126 | (86) |  |
| High (= 9) | 151 | 112 | (74) |  |  | 79 | (52) |  |  | 62 | (41) |  |  | 122 | (81) |  |  | 71 | (47) |  |  | 113 | (75) |  |
| CDC25B nucleus^2^ |  |  |  | 0.17 |  |  |  | 0.04 |  |  |  | 0.53 |  |  |  | 0.65 |  |  |  | 0.43 |  |  |  | 0.16 |
| Low (≤ 6 ) | 251 | 195 | (78) |  |  | 138 | (55) |  |  | 124 | (49) |  |  | 217 | (87) |  |  | 132 | (53) |  |  | 205 | (82) |  |
| High (> 6) | 47 | 41 | (87) |  |  | 34 | (72) |  |  | 26 | (55) |  |  | 39 | (83) |  |  | 28 | (60) |  |  | 34 | (72) |  |
| CDC25C cytoplasm^2^ |  |  |  | 0.38 |  |  |  | 0.002 |  |  |  | 0.001 |  |  |  | 0.005 |  |  |  | <0.001 |  |  |  | 0.13 |
| Low (≤ 3) | 110 | 84 | (76) |  |  | 50 | (46) |  |  | 41 | (37) |  |  | 86 | (78) |  |  | 42 | (38) |  |  | 83 | (75) |  |
| High (> 3) | 188 | 152 | (81) |  |  | 122 | (65) |  |  | 109 | (58) |  |  | 170 | (90) |  |  | 118 | (63) |  |  | 156 | (83) |  |
| Phospho-CDC25C (Ser216) cytoplasm^2^ |  |  |  | 0.78 |  |  |  | 0.003 |  |  |  | 0.08 |  |  |  | 0.007 |  |  |  | <0.001 |  |  |  | 0.37 |
| Low (≤ 3) | 147 | 115 | (78) |  |  | 72 | (49) |  |  | 66 | (45) |  |  | 118 | (80) |  |  | 63 | (43) |  |  | 121 | (82) |  |
| High (>3) | 151 | 121 | (80) |  |  | 100 | (66) |  |  | 84 | (56) |  |  | 138 | (91) |  |  | 97 | (64) |  |  | 118 | (78) |  |
| Phospho-CDC25C (Ser216) nucleus^2^ |  |  |  | 0.007 |  |  |  | 0.30 |  |  |  | <0.001 |  |  |  | 0.002 |  |  |  | 0.61 |  |  |  | 0.007 |
| Low (−) | 87 | 60 | (69) |  |  | 46 | (53) |  |  | 27 | (31) |  |  | 66 | (80) |  |  | 49 | (56) |  |  | 61 | (70) |  |
| High (+) | 211 | 176 | (83) |  |  | 126 | (60) |  |  | 123 | (58) |  |  | 190 | (90) |  |  | 111 | (53) |  |  | 178 | (84) |  |
| 14-3-3σ cytoplasm^2^ |  |  |  | 0.02 |  |  |  | <0.001 |  |  |  | <0.001 |  |  |  | <0.001 |  |  |  | <0.001 |  |  |  | 0.15 |
| Low (< 6) | 83 | 58 | (70) |  |  | 31 | (37) |  |  | 23 | (28) |  |  | 59 | (71) |  |  | 30 | (36) |  |  | 62 | (75) |  |
| High (≥ 6) | 215 | 178 | (83) |  |  | 141 | (66) |  |  | 127 | (59) |  |  | 197 | (92) |  |  | 130 | (61) |  |  | 177 | (82) |  |
| 14-3-3σ nucleus^2^ |  |  |  | 0.67 |  |  |  | 0.15 |  |  |  | 0.03 |  |  |  | 0.24 |  |  |  | 0.41 |  |  |  | 0.002 |
| Low (< 6) | 122 | 95 | (78) |  |  | 64 | (53) |  |  | 52 | (43) |  |  | 101 | (83) |  |  | 62 | (51) |  |  | 87 | (71) |  |
| High (≥ 6) | 176 | 141 | (80) |  |  | 108 | (61) |  |  | 98 | (56) |  |  | 155 | (88) |  |  | 98 | (58) |  |  | 152 | (86) |  |
| 14-3-3β cytoplasm |  |  |  | - |  |  |  | < 0.001 |  |  |  | 0.001 |  |  |  | 0.01 |  |  |  | 0.04 |  |  |  | 0.86 |
| Low (≤ 1) | 62 | - | - |  |  | 22 | (36) |  |  | 19 | (31) |  |  | 47 | (76) |  |  | 26 | (42) |  |  | 49 | (79) |  |
| High (>1) | 236 | - | - |  |  | 150 | (64) |  |  | 131 | (56) |  |  | 209 | (89) |  |  | 134 | (57) |  |  | 190 | (81) |  |
| 14-3-3γ cytoplasm |  |  |  | <0.001 |  |  |  | - |  |  |  | <0.001 |  |  |  | 0.001 |  |  |  | <0.001 |  |  |  | 0.38 |
| Low (≤ 3) | 126 | 86 | (68) |  |  | - | - |  |  | 36 | (29) |  |  | 98 | (78) |  |  | 45 | (36) |  |  | 98 | (78) |  |
| High (>3) | 172 | 150 | (87) |  |  | - | - |  |  | 114 | (66) |  |  | 158 | (92) |  |  | 115 | (67) |  |  | 141 | (82) |  |
| 14-3-3ζ cytoplasm |  |  |  | 0.001 |  |  |  | <0.001 |  |  |  | - |  |  |  | 0.003 |  |  |  | 0.001 |  |  |  | 0.11 |
| Low (≤ 3) | 148 | 105 | (71) |  |  | 58 | (39) |  |  | - | - |  |  | 118 | (80) |  |  | 65 | (44) |  |  | 113 | (76) |  |
| High (>3) | 150 | 131 | (87) |  |  | 114 | (76) |  |  | - | - |  |  | 138 | (92) |  |  | 95 | (63) |  |  | 126 | (84) |  |
| 14-3-3ε cytoplasm |  |  |  | - |  |  |  | - |  |  |  | - |  |  |  | - |  |  |  | <0.001 |  |  |  | 0.84 |
| Low (≤ 1) | 42 | - | - |  |  | - | - |  |  | - | - |  |  | - | - |  |  | 7 | (17) |  |  | 33 | (79) |  |
| High (>1) | 256 | - | - |  |  | - | - |  |  | - | - |  |  | - | - |  |  | 153 | (60) |  |  | 206 | (80) |  |
| 14-3-3η cytoplasm |  |  |  | - |  |  |  | - |  |  |  | - |  |  |  | <0.001 |  |  |  | - |  |  |  | 1 |
| Low (≤ 3) | 138 | - | - |  |  | - | - |  |  | - | - |  |  | 103 | (75) |  |  | - | - |  |  | 111 | (80) |  |
| High (>3) | 160 | - | - |  |  | - | - |  |  | - | - |  |  | 153 | (96) |  |  | - | - |  |  | 128 | (80) |  |
| 14-3-3τ nucleus |  |  |  | - |  |  |  | - |  |  |  | - |  |  |  | 0.84 |  |  |  | 1 |  |  |  | - |
| Low (≤ 3) | 239 | - | - |  |  | - | - |  |  | - | - |  |  | 206 | (86) |  |  | 128 | (54) |  |  | - | - |  |
| High (>3) | 59 | - | - |  |  | - | - |  |  | - | - |  |  | 50 | (85) |  |  | 32 | (54) |  |  | - | - |  |

^1^ Pearson chi-square

^2^ In previous reports, 14-3-3σ and CDC25s have been studied in the same cohort of vulvar carcinomas [16,17]. CDC25A and CDC25B expression were observed only in nucleus, whereas CDC25C expression was identified only in cytoplasm.

H = High

L = Low
